# Body mass index and obstetric outcomes in Saudi Arabia: a prospective cohort study

**DOI:** 10.4103/0256-4947.67075

**Published:** 2010

**Authors:** Abdel-Hady El-Gilany, Sabry Hammad

**Affiliations:** a College of Medicine, King Faisal University, Al-Hassa, Saudi Arabia; b Ministry of Health, Riyadh, Saudi Arabia

## Abstract

**BACKGROUND AND OBJECTIVES:**

We examined the effect of body mass index in early pregnancy on pregnancy outcome since no study in Saudi Arabia has addressed this question.

**METHODS:**

This prospective cohort study involved women registered for antenatal care during the first month of pregnancy at primary health care centers in Al-Hassa, Saudi Arabia. Data was collected from records and by direct interview.

**RESULTS:**

The study included 787 women. Compared to normal weight women (n=307), overweight (n=187) and obese (n=226) women were at increased risk for pregnancy-induced hypertension (RR=4.9 [95% CI 1.6-11.1] and 6.1 [95% CI 2.1-17.8], respectively), gestational diabetes (RR=4.4 [95% CI 1.2-16.3] and 8.6 [95% CI 2.6-28.8]), preeclamptic toxemia (RR=3.8 [95% CI 1.1-14.6] and 5.9 [95% CI 1.7-20.4]), urinary tract infections (RR=1.4 [95% CI 0.5-3.9] and 3.7 [95% CI 1.7-6.2]), and cesarean delivery (RR=2.0 [95% CI 1.3-3.0] in obese women). Neonates born to obese women had an increased risk for postdate pregnancy (RR=3.7 [95% CI 1.2-11.6]), macrosomia (RR=6.8 [95% CI 1.5-30.7]), low 1-minute Apgar score (RR=1.9 [95% CI 1.1-3.6]), and admission to neonatal care units (RR=2.1 [95% CI 1.2-2.7]). On the other hand, low birth weight was less frequent among obese women (RR=0.5 [95% CI 0.3-0.9]) while the risk was high among underweight women (RR=2.3 [95% CI 1.4-3.8])

**CONCLUSION:**

Even with adequate prenatal care, overweight and obesity can adversely affect pregnancy outcomes.

Women who are overweight or obese before they become pregnant have a higher risk for complications during pregnancy and can expect more problems for the children.^1^ This is especially troubling in light of the current obesity epidemic.^1^ The most consistently described maternal complications during pregnancy and delivery in obese women are gestational diabetes, pregnancy-induced hypertension and preeclampsia, venous thromboembolism, the necessity for labor induction, and cesarean delivery.^2^–^10^ Maternal overweight and obesity are associated with a greater risk of stillbirth, perinatal death, preterm delivery, fetal macrosomia, fetal birth defects, and admission to neonatal intensive care.^3^,^7^,^10^,^11^ A recent study revealed that 31.5% of Saudi females of childbearing age are overweight and 21.1% are obese.^12^

The effect of maternal underweight on obstetric performance is less clear. While some researchers have found an increased incidence of preterm delivery, low birth weight, and perinatal loss in these women,^13^,^14^ others have reported a protective effect of maternal underweight on certain pregnancy complications and interventions.^15^ Despite these alarming levels of overweight and obesity, to the best of our knowledge no study on the effects of body mass index on pregnancy outcomes has been done in Saudi Arabia. Therefore, this study aimed to examine the association between early pregnancy body mass index and adverse maternal and neonatal outcomes.

## METHODS

This prospective study was carried out in Al-Hassa, Saudi Arabia. The target population was women initiated into antenatal care in the first month of pregnancy during the year 2007. Al-Hassa is the largest province in Saudi Arabia’s eastern region, having a population of 908366. Maternity care in Al-Hassa is provided through a network of 47 primary health care centers (PHCCs) that cover urban, rural, and hegar (Bedouin desert collection) areas. In addition, there are facilities provided by the private sector, ARAMCO Petroleum Company, and the National Guard and Maternity Hospital. Antenatal care was by the classic schedule, with 13 visits throughout pregnancy.

Eligible candidates were all women attending PHCCs for antenatal care within the first month of pregnancy and willing to come for regular follow-up throughout pregnancy. Exclusion criteria were any pre-pregnancy chronic medical disease (e.g., hypertension, diabetes, renal or cardiac disease, and sickle cell disease) and multiple pregnancies. Women were counseled and assured that data collected would be kept confidential. All subjects gave verbal consent before the interview. At the time of the study there was no institutional review board at the College of Medicine in Al-Hassa and so approval for the study was obtained from the Directorate of Health, Al-Hassa.

Over the year (2007), a total of 1089 women in the first month of pregnancy registered for antenatal care. These represented 13.1% of all women registered for antenatal care at PHCCs during the study period. Gestational age was assessed by self-reported last menstrual period. Of the 1089 women, 791 were included in the study. Details on the calculation of sample size and sampling methodology, as well as the exclusion criteria applied and causes for refusal to participate, have been reported in a previous article on the prevalence of obesity in this group of women.^16^ The women were followed up during their routine antenatal visits.

The women were interviewed at the PHCCs by Arabic-speaking female nurse interviewers who were oriented to the study and trained on data collection. A predesigned and tested questionnaire was used for the interview. Data were collected from the family file and the maternity cards maintained at the PHCCs and also from the hospital discharge form. The Ministry of Health had developed special guidelines for the maintenance of the maternity card, with clear explanations of its contents and of how it is to be used; the various measurements and investigations were also described. The card was shared by the health centers and the hospital. There was continuous stress on the need to keep this card complete and up-to-date.^17^

The height (in meters) of the study participants was recorded at the first antenatal visit, while the weight (in kilograms) was recorded at each visit. Height was measured with a stadiometer accurate to 0.1 cm, with the mother standing and without shoes. Body weight was measured with a calibrated electronic Seca scales (Seca Ltd, Birmingham, UK) accurate to 0.1 kg, with the subject wearing the lightest possible clothes. The measurements were used to calculate Quetelet’s index or the body mass index (BMI) using the formula weight (in kg)/(height in meters)^2^. According WHO, BMI values are classified into four categories: underweight: <18.5, normal weight: 18.5-24.99, overweight: 25-29.9, and obese ≥30.^18^–^20^

The obstetric outcomes that we examined included pregnancy-induced hypertension, gestational diabetes mellitus, antepartum hemorrhage, preeclamptic toxemia, anemia, urinary tract infections, route of delivery, gestational age at delivery, stillbirth, birth weight, 1-min Apgar score, and admission to a neonatal care unit. Gestational age at birth was defined as the number of completed weeks of gestation based on the delivery date in the clinical record. The definitions employed were as follows: a preterm delivery was a live infant delivered at <37 weeks’ gestation, a postdate delivery was a live infant delivered at >42 weeks’ gestation, low birth weight (LBW) was a live infant weighing <2500 g at birth, and macrosomia referred to a live infant weighing >4000 g at birth.^21^,^22^

Demographic, antenatal, and natal data were examined. Women with normal body weight were used as the reference or comparison group for the analysis. The unpaired *t* test was used for comparison of quantitative variables. The chi-square test was used as a test of significance for comparison of categorical variables. P≤.05 was chosen as the level of statistical significance. We used the SPSS v. 11 (Chicago, USA) for the statistical analysis. To quantify the risk of bad antenatal or neonatal outcomes in the study groups, we calculated the relative risk (RR) with the 95% confidence intervals (CI).

## RESULTS

Four women were excluded from the final analysis (two had abortions, one had a twin pregnancy, and one was lost to follow-up) and therefore data on 787 women were available for analysis at the end of the study.

Underweight women were of younger age and of lower gravidity compared to normal weight women, while obese women were of older age and of higher gravidity than normal weight women (Table 1). Compared to normal weight women, overweight and obese women were at increased risk for pregnancy-induced hypertension, gestational diabetes, preeclamptic toxemia, urinary tract infections and for cesarean delivery (Table 2). Neonates born to an obese mother had an increased risk of postdate pregnancy, macrosomia, low 1-min Apgar score, and admission to neonatal care units (Table 3). On the other hand, low birth weight was less frequent among obese women and more common among underweight women. No maternal deaths were recorded in our study population.

**Table 1:** Maternal sociodemographic and anthropometric characteristics.

	Normal (307)(BMI 18.5–24.99)	Underweight (67)(BMI <18.5)	Overweight (187)(BMI ≥25-<30)	Obese (226)(BMI ≥30)
Age (mean [SD])	25.9 (5.9)	24.1 (5.4)	28.8 (6.3)^a^	30.7 (6.4)^a^
Antenatal visits (mean [SD])	8.2 (2.3)	8.5 (2.6)	8.1 (2.5)	8.1 (2.6)
Gravidity (mean [SD])	2.8 (2.4)	2.2 (1.9)	3.8 (2.7)^a^	5.2 (3.4)^a^
Less than secondary education n (%)	119 (38.8)	24 (35.8)	82 (43.9)	106 (46.9)
Housewives n (%)	250 (81.4)	56 (83.6)	155 (82.9)	190 (84.1)
Unsatisfactory family income n (%)	81 (26.4)	18 (26.8)	51 (27.3)	59 (26.1)
Early pregnancy weight (kg) (mean [SD])	52.9 (6.0)	42.4 (3.3)^a^	66.0 (5.6)^a^	83.7 (11.9)^a^
Height (cm) (mean [SD])	155.7 (5.4)	156.0 (5.2)	155.0 (5.2)	155.1 (6.2)
Hospital delivery n (%)	206 (99.7)	66 (97.0)	186 (99.5)	223 (98.7)

a *P*≤.001 versus normal weight.

**Table 2:** Maternal prenatal morbidities and mode of delivery.

	Normal (BMI 18.5-24.99) (n=307)	Underweight (BMI<18.5) (n=67)	Overweight (BMI≥25—29.99) (n=187)	Obese (BMI ≥30) (n=226)
Pregnancy-induced hypertension	4 (1.3)	0	12 (6.4)4.9 (1.6-11.1)^a^	18 (8.0)6.1 (2.1-17.8)^b^
Gestational diabetes mellitus	3 (1.0)	0	8 (4.2) 4.4 (1.2-16.3)^c^	19 (8.4) 8.6 (2.6-28.8)^b^
Antepartum hemorrhage	3 (1.0)	0	1 (0.5) 0.6 (0.1-5.2)	5 (2.2) 2.3 (0.6-9.4)
Preeclamptic toxemia	3 (1.0)	0	7 (3.7) 3.8 (1.1-14.6)^c^	13 (5.8) 5.9 (1.7-20.4)^a^
Urinary tract infections	8 (2.6)	3 (4.5) 1.7 (0.5-6.3)	7 (3.7) 1.4 (0.5-3.9)^a^	22 (9.7) 3.7 (1.7-6.2)^a^
Anemia (Hb<10.5 g%)	145 (47.2)	33 (49.2) 1.0 (0.8-1.4)	75 (40.1) 0.9 (0.7-1.1)	103 (45.6) 1.0 (0.8-1.2)
Cesarean delivery	30 (9.8)	4 (6.0) 0.6 (0.2-1.7)	21 (11.2) 1.2 (0.6-2.0)	43 (19.0) 2.0 (1.3-3.0)^a^
Other problems*	8 (2.6)	2 (3.0) 1.2 (0.3-5.3)	3 (1.6) 0.6 (0.2-2.3)	7 (3.1) 1.2 (0.4-3.2)

Values are n (%) and relative risk and 95% confidence interval except for reference group.

* Hyperemesis, oligohydraminos, polyhydraminos, and incompetent cervix.

a *P*≤.01 versus normal weight;

b *P*≤.001 versus normal weight;

b *P*≤.05 versus normal weight.

**Table 3:** Neonatal outcomes.

	Normal (BMI 18.5-24.99) (n=307)	Underweight (BMI<18.5) (n=67)	Overweight (BMI≥25—29.99) (n=187)	Obese (BMI ≥30) (n=226)
Stillbirth	2 (0.7)	1 (1.5) 2.3 (0.2-24.3)	2 (1.1) 1.6 (0.2-11.5)	2 (1.1) 1.4 (0.2-9.6)
Preterm delivery (<37 weeks)	19 (6.3)	6 (9.6) 1.5 (0.6-3.5)	10 (5.4) 0.9 (0.4-1.8)	13 (5.8) 0.9 (0.5-1.8)
Postdate (>42 weeks)	4 (1.4)	2 (3.0) 2.3 (0.4-12.3)	5 (2.7) 2.0 (0.6-7.1)	11 (4.9) 3.7 (1.2-11.6)^a^
Male baby	162 (52.8)	34 (50.7) 1.0 (0.7-1.3)	97 (51.9) 1.0 (0.8-1.2)	117 (51.7) 1.0 (0.8-1.2)
Low birth weight (<2.5 kg)	36 (11.7)	18 (26.9) 2.3 (1.4-3.8)^b^	18 (9.6) 0.6 (0.2-1.6)	134 (5.8) 0.5 (0.3-0.9)^a^
Macrosomia (>4 kg)	2 (0.7)	0	4 (2.1) 3.3 (0.6-17.8)	10 (4.4) 6.8 (1.5-30.7)^c^
One-minute Apgar score <7	15 (4.9)	4 (6.0) 1.2 (0.4-3.6)	11 (5.8) 1.2 (0.6-2.6)	21 (9.3) 1.9 (1.1-3.6)^a^
Admission to neonatal intensive care	18 (5.9)	5 (7.5) 1.3 (0.5-3.3)	18 (9.6) 1.6 (0.9-3.1)	28 (12.4) 2.1 (1.2-2.7)^b^

Values are n (%) and relative risk and 95% confidence interval except for reference group.

a *P*≤.05 versus normal weight;

b *P*≤.01 versus normal weight;

c *P*≤.001 versus normal weight.

## DISCUSSION

The impact of increased BMI in the general population has been the focus of many studies, but studies pertaining to pregnant women are few.^23^ What studies have been reported have all been from Western countries; there are no Saudi studies on pregnant women. The findings of Western studies may not apply to the Saudi population.

Previous studies have found that low maternal BMI increases obstetric risk. The complications include maternal anemia, preterm labor, intrauterine growth retardation, and low birth weight.^23^–^26^ In the present study, a significant association between low maternal weight and low birth weight was seen. On the other hand, no association was found between low maternal weight and anemia, which was probably because the study population was adequately covered with antenatal care and receiving routine iron and folic acid supplementation. We found that obese women were at increased risk of pregnancy-induced hypertension, gestational diabetes mellitus, preeclampsia, urinary tract infection, cesarean delivery, postdate pregnancy, and macrosomia. Furthermore, babies born to obese mothers had an increased risk for low Apgar score at birth and admission to intensive neonatal care units. These findings are consistent with other studies.^2^–^11^,^24^,^27^,^28^

In our study, the relative risk of pregnancy-induced hypertension was found to be 4.9 and 6.1 among overweight and obese women, respectively. Previous studies have reported risks of 1.7 and 1.9 among overweight women, while among the obese the risk ranged from 1.2 to 4.8.^3^,^6^,^8^,^27^ In this study, the relative risk of gestational diabetes was 4.4 and 6.1 among overweight and obese women, respectively. Previous studies have reported risks of 1.7 and 1.8 among overweight women, while the risk ranged from 3.0 to 15.3 among obese women. 3,8,10 The relative risk for preeclamptic toxemia in our study population was 3.8 for overweight women and 5.9 for obese women. Previous studies reported risks ranging from 1.1 to 2.7. 6,8,10 The relative risk for urinary tract infection was 1.4 and 3.7 in overweight and obese women, respectively; the corresponding figures in a previous study were 1.2 and 1.4.^10^ The risk for cesarean delivery was doubled in obese women as compared to normal weight women in our study, whereas previous studies reported a similar risk for cesarean section in these two groups.^3^,^7^–^10^

The relative risk for postdate pregnancy was 3.7 among obese women in our study; this is higher than the relative risk of 2 reported in a previous study.^5^ Macrosomia was about 7 times more likely among obese women than in normal weight women, which is much higher than the relative risk of 2.4 reported in an earlier study.^10^ The risk for low 1-min Apgar score was nearly double among neonates of obese women as compared to the babies born to mothers of normal weight; this is slightly more than the relative risk of 1.6 reported in a previous study.^7^ On the other hand, one study reported no difference in Apgar score between neonates of obese and normal weight women.^8^ In our study, the neonates of obese women were two times more likely to be admitted to intensive care units than those of normal weight women, which agrees with the findings of one earlier study.^7^ A much higher risk (RR=7.14) was reported by Callaway et al,^3^ while Rode et al^8^ reported no difference. The risk for low birth weight birth was lower among obese women compared to normal weight women (RR=0.5). Bhattacharya et al^25^ found a strong positive association of birth weight with maternal BMI.

It is not clear whether obesity is a direct cause of adverse pregnancy outcome or whether the association between obesity and adverse outcome is due to factors or characteristics that are shared by both entities, such as advanced maternal age, higher gravidity and associated pregnancy complications. No randomized trials have been performed to investigate this relationship; however, indirect data suggest a possible causal association.

This study adds to the increasing evidence suggesting that obesity during pregnancy is associated with numerous maternal and perinatal risks. Managing these problems and reducing their occurrence can pose a challenge to obstetrical care providers. Hui et al^29^ revealed the feasibility of lifestyle interventions (physical exercise and diet) during pregnancy and its potential to improve pregnancy outcomes. Although the obese women included in our study were receiving adequate antenatal care, they experienced many adverse pregnancy outcomes. Health education to control body weight before pregnancy is warranted. Obese women should consider losing weight through diet modification and exercise before becoming pregnant. They should continue exercising and keep a close watch on their weight gain during pregnancy and should consider consulting a dietitian when necessary. A nationwide community-based prospective study should provide in-depth knowledge about the prevalence and impact of different categories of BMI on pregnancy outcomes among different groups.

A limitation of the study is that it was clinic-based and included women attending PHCCs in only one region of Saudi Arabia. Late attendees, those who received care in other health sectors, and those receiving no care at all were not included. Weight and height were measured at the booking antenatal visit during the first month of pregnancy; pre-pregnancy measurements were not available. However, it has been reported that gestational BMI had similar predictive value as pre-pregnancy BMI, as shown by similar areas under the receiver operating characteristic curves in those studies.^30^
